# Mortality of Suicide and Cerebro-Cardiovascular Diseases by Occupation in Korea, 1997–2020

**DOI:** 10.3390/ijerph191610001

**Published:** 2022-08-13

**Authors:** Jungwon Jang, Inah Kim

**Affiliations:** 1 Institute for Health and Society, Hanyang University, Seoul 04763, Korea; 2 Department of Occupational and Environmental Medicine, College of Medicine, Hanyang University, Seoul 04763, Korea

**Keywords:** suicide, cerebro-cardiovascular diseases, indirect standardized mortality ratio, proportional mortality ratio, overwork-related death, socioeconomic status, upper non-manual worker, female manager, occupation, economic crisis

## Abstract

Although studies on occupational mortality have been conducted in Korea, the results for occupations with high mortality around 2010 are inconsistent. This study aimed to examine occupational mortality from overwork-related suicide and cerebro-cardiovascular diseases (CCVD) from 1997 to 2020. We used microdata of the Causes of Death Statistics (CDS) and Economically Active Population Survey (EAPS) to obtain indirect standardized mortality ratio (SMR) and standardized proportional mortality ratio (PMR) of suicide (X60–X84) and CCVD deaths (I20–I25 and I60–I69) by gender and eight occupational categories. The trend of SMR of suicide and CCVD by occupation was similar within individual genders. The SMR of managers (MNG) was the highest for men and women in 2012–2017 and 2008–2020, respectively, whereas the SMR of professionals and related workers (PRF) was consistently low. Despite the similar socioeconomic status of MNG and PRF, we suggest that their mortality should be analyzed separately in Korea. SMR of suicide and CCVD in female MNG were consistently highest, although the PMR was low. Female MNG may have been more directly affected by the economic crisis. There is a need for work-related stress management, early intervention, and prevention policies in occupations vulnerable to mortality.

## 1. Introduction

Suicide and cerebro-cardiovascular diseases (CCVD) are the leading cause of death globally and are known to be influenced by a complex interaction between various factors [1,2]. In particular, suicide is not only influenced by economic problems, health problems, and personally stressful environments but also work-related stresses [1]. Some East Asian countries, such as Taiwan, Japan, and South Korea, consider suicide and CCVD overwork-related diseases and provide compensation for death [3,4,5,6]. Death by CCVD and suicide have been found to be related to long working hours and various work-related stresses in Korea [7,8,9]. 

Mortality from overwork-related diseases has been reported to vary by occupation, which is an important factor affecting health [10]. In general, occupational mortality was closely related to socioeconomic status. Although the classification of occupation differed by study, in most Western countries, suicide and CCVD mortality rates continued to be higher in unskilled occupations with low socioeconomic status than for managers and professionals with high socioeconomic status [11,12,13,14,15,16]. This may be the effect of social inequality in various areas such as income, education, and medical service [17]. However, Japan is known to be a representative country with higher mortality for upper non-manual workers (managers and professionals) after the economic bubble burst [18,19], whereas the results for Korea are conflicting. In recent years, studies have reported that the mortality of upper non-manual workers was higher than that of manual workers [9,19], whereas others found it to be lower [20,21]. The discrepancy may be because of a numerator–denominator bias due to differences in the use of unlinked data [20]. However, studies using the same unlinked data showed different results for suicide mortality rates, especially for managers [9,21]. Furthermore, although suicide and CCVD may seem heterogeneous, some similarities regarding mortality can be inferred from previous studies [9,15,18,19,20]. However, few studies have analyzed the mortality trend of suicide and CCVD over time using long-term data in Korea.

This study aimed to analyze the mortality trends of suicide and CCVD by occupation among people aged 25–64 years from 1997 to 2020 in Korea.

## 2. Materials and Methods

### 2.1. Data Sources

The indirect standardized mortality ratio (SMR) and standardized proportional mortality ratio (PMR) of suicide and CCVD deaths by gender and occupation for 1997–2020 were calculated using microdata by Statistics Korea [22] from the Causes of Death Statistics (CDS) and Economically Active Population Survey (EAPS). 

CDS provided cause of death (International Classification of Diseases 10th Revision (ICD-10)), age at death, gender, and occupation. Suicide was classified as intentional self-harm (ICD–10 code: X60–X84), and CCVD as ischemic heart diseases (I20–I25) and cerebrovascular diseases (I60–I69). The CDS category recording the deceased’s occupation was changed from “occupation before death” to “occupation at the time of illness or accident” around 2000. After 2018, as occupation was excluded from the death certificate, Statistics Korea obtained the occupation of the deceased from administrative data (such as employment insurance and employee DB).

EAPS provided age, gender, occupation, and economic activity status. EAPS divides the working-age population into economically active and inactive populations. The economically active population is divided into employed and unemployed people. Employed people listed on EAPS were used as a reference population for expected death events when calculating SMR.

As unemployed people have various risk factors and are known to be associated with an increased risk of death [16,18], both the study and the reference population used only the employed. Employed people include wage workers (regular, non-permanent, part-time, and non-typical workers) and non-wage workers (self-employed persons). Occupational categories were selected from the 7th version of the Korean Standard Classification of Occupation (KSCO). The classification of occupation for the 5th and 6th versions of CDS and EAPS was changed to that of the 7th KSCO for the analysis. CDS provided data for “Service and Sales Workers” by integrating “Service workers” and “Sales workers” into the same occupational categories. Therefore, we consolidated EAPS data into “Service and Sales Workers” according to the CDS classification. Since EAPS only provides the 4th version of KSCO for 1997–1999, EAPS for 1997–1999 used 2000 data for consistency of classification with the CDS. Additionally, Armed Forces were excluded from the analysis because EAPS does not include Armed Forces in occupation categories. That is, the eight occupational categories used in the study were managers (MNG); professionals and related workers (PRF); clerks (CLK); service and sales workers (SSL); skilled agricultural, forestry, and fishery workers (SFF); craft and related trade workers (CRT); equipment, machine operating, and assembling workers (QMS); and elementary workers (LMT). 

The study population was limited to those aged 25–64 years old, excluding those aged 15–24. It was not possible to calculate SMR and PMR by stratifying the age by five years because suicide and CCVD deaths and population by occupation among 15–24-year-olds were zero or very small in number.

### 2.2. Ethics Statement

Both CDS and EAPS provided microdata without personal identification information. This study was approved by the Institutional Review Board at Hanyang University, Seoul, Republic of Korea (HYUIRB–202012–005–2).

### 2.3. Statistical Analysis

SMR can be calculated even when the number of deaths is small and variance is relatively low [23]. In general, the economically active population (including employed and unemployed people) is used as the reference population. However, as mentioned above, in order to avoid the effect of the death of the unemployed [16,18], only the deaths among employed people were considered the target population. Since SMR may have denominator–numerator bias due to unlinked data to the reference population and deaths, PMR was used to overcome this bias. 

SMR is calculated by comparing the actual number of suicide and CCVD deaths with the expected number of suicide and CCVD deaths in the reference population. PMR is calculated by comparing suicide and CCVD deaths among total deaths in the study group with suicide and CCVD deaths among total deaths in the reference group. We stratified the age by five years for adjustment and analyzed the data by gender and occupation with estimated 95% confidence intervals (95% CI). All statistical procedures for SMR and PMR were performed using SAS software version 9.4 (SAS Institute Inc., Cary, NC, USA) and R software version 4.2.0 [24].

## 3. Results

**Figure 1:**
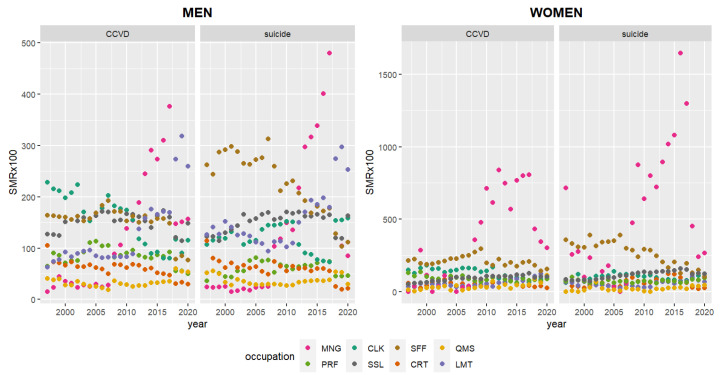
Annual indirect standardized mortality ratio (SMR) of suicide and CCVD in 1997–2020. CCVD—cerebro-cardiovascular disease; MNG—managers; PRF—professionals and related workers; CLK—clerks; SSL—service and sales workers; SSF—skilled agricultural, forestry, and fishery workers; CRT—craft and related trade workers; QMS—equipment, machine operating, and assembling workers; LMT—elementary workers.

Figure 1 shows similar trends in annual SMR of suicide and CCVD within individual genders from 1997 to 2020. Namely, male suicide SMRs are similar to male CCVD SMRs, while female suicide SMRs are the same as female CCVD SMRs. Male CCVD and suicide had the highest SMRs for CLK and SFF in 1997–2011, MNG in 2012–2017, and LMT in 2018–2020. Female CCVD and suicide had the highest SMR for SFF in 1997–2007 (excluding suicide SMR in 1997, when MNG was highest), and for MNG in 2008–2020.

Table 1 shows male suicide SMR and PMR by occupation every three years from 1997 to 2020. The SMR of male SFF was the highest for 15 years from 1997 to 2011 and the highest in 2000–2002 (SMR 2.90, 95% CI 2.77–3.04). Since 2012–2014, the SMR of SFF gradually decreased. The PMR of male SFF was also high during 1997–2008, with the highest in 2000–2002 (PMR 1.17, 95% 1.11–1.22). SMR of MNG was highest in 2015–2017 (SMR 4.01, 95% CI 3.76–4.26). Prior to 2012, the PMR of MNG was low or not significant. However, in 2012–2017, it was the second highest after SSL. In 2018–2020, LMT and CLK had the highest SMR (2.75, 95% CI 2.65–2.85) and PMR (1.35, 95% CI 1.30–1.40), respectively. The suicide SMR and PMR of SSL were consistently high over 24 years (except for PMR in 2000–2002). Conversely, both SMR and PMR were consistently low for CRTs for 24 years.

SMR of female suicide was the highest for MNG and SFF (Table 2). Female SFF had the highest SMR in 2000–2008 and the highest PMR in 2000–2005. After the highest SMR of female MNG (3.89, 95% CI 1.69–6.10) in 1997–1999, it increased greatly again in 2009–2020. SMR was the highest at 13.17 (95% CI 10.55–15.79) in 2015–2017. Conversely, the PMR of female MNG was low or not significant for 24 years.

Male CLK had the highest SMR of CCVD than other occupations in 1997–2011, especially in 1997–1999 (SMR 2.19, 95% CI 2.09–2.29), and their PMR was consistently higher during the same period (Table 3). The CCVD SMR of male MNG was the highest in 2012–2017, especially in 2015–2017 (SMR 3.16, 95% 2.95–3.37). Their PMR was the highest for most of the periods (2000–2005, 2009–2014, and 2018–2020). LMT had the highest SMR (2.83, 95% CI 2.73–2.94) in 2018–2020. The CCVD SMR and PMR of SSL were consistently high for 24 years.

The CCVD SMR of females was the highest for SFF in 1997–2008, followed by CLK, whereas the PMR of both occupations was low or insignificant (Table 4). After 2009–2011, the CCVD SMR of female MNG rose dramatically. In particular, the SMR of MNG was the highest at 7.93 (95% CI 5.89–9.97) in 2015–2017. Conversely, the PMR of female MNG was low or not significant for 24 years.

Over the 24-year period, SMR of suicide and CCVD were highest in SFF and MNG among males and females, respectively (Appendix A). Appendix B displays the result of analyzing the SMR of male suicide and CCVD in 2007–2018 by period and four occupation groups, simila r to the recent linked data study [20]. As a result of sorting them into four occupational groups, the SMR of upper non-manual workers (MNG and PRF) was lower than that of other groups. However, as a result of analyzing MNG and PRF separately in eight groups, the SMR of MNG increased to first (CCVD) and second (suicide).

## 4. Discussion

The mortality ratios of suicide and CCVD were identified according to eight occupational categories and gender in 1997–2020. Interestingly, the suicide and CCVD mortality ratios by occupation showed similar trends within individual genders, with marked changes observed in 2012 for men and 2008 for women. In men, SFF in suicide and CLK in CCVD had the highest SMR in 1997–2011. Both suicide and CCVD SMRs were the highest for MNG in 2012–2017. Additionally, for both suicide and CCVD in 2018–2020, LMT had the highest SMR but lower PMR. Suicide and CCVD SMR of SFF were highest among women in 2000–2008. Both female MNG’s suicide and CCVD had the highest SMR in 2009–2020, but the PMR was low or not significant. Japanese studies also showed similar patterns of cardiovascular disease, suicide, and cancer mortality in the same occupation [18,19]. Large prospective studies found that depression and psychological distress were common risk factors for an increase in all-cause mortality, including cardiovascular disease and suicide mortality [25,26]. CCVD may induce suicide by various psychopathological means [27]. However, studies on the relationship between overwork-related diseases are scarce. The CCVD SMR of CLK was the highest in 1997–2011 but only slightly higher in suicide for the same period. CLK, a typically sedentary job, is known to have a higher risk of CCVD [28].

Suicide and CCVD mortality studies in various European countries [11,15,16], the United States [13], Canada [14], and New Zealand [12] reported that MNG and PRF had the lowest risk of suicide, but manual workers such as LMT, QMS, and SFF had a high risk. Japanese studies, however, found that workers with high socioeconomic status, including MNG and PRF, had higher mortality risks of ischemic heart disease, cerebrovascular disease, and suicide after the economic bubble burst [18,19,29]. In contrast, the results of studies on occupational mortality in Korea are inconsistent. Some studies using unlinked data showed that male mortality of upper non-manual workers (MNG and PRF) turned out to be higher than that of manual workers in the mid-2010s, unlike in the late 1990s [9,19], whereas others reported that the mortality of manual workers remained the highest after the late 2000s [20,21].

It has been argued that one of the reasons for this discrepancy in the results may be due to differences in the data used for mortality analysis [20]. Numerator–denominator bias occurs when death and census data are not linked, which is known to have the potential to influence mortality estimates [30]. However, the effect and direction of the numerator–denominator bias are still controversial. Similar results were found with linked data from 1995 to 2008 [31] and unlinked data from 1995 to 1999 [19], with the lowest mortality for MNG and PRF. Additionally, studies using the same unlinked data reported different results in MNG suicide mortality [9,21].

Another reason may be that MNG and PRF were not analyzed separately in previous studies. A previous Korean study reported a consistently lower suicide mortality rate for MNG during 1993–2016 [21]. However, this result may be because MNG and PRF were included in the same group. The present findings show that the SMR of MNG has rapidly increased since around 2010, whereas the SMR of PRF has been consistently low. To clarify this, we compared the SMR of eight occupational groups by sorting them into four occupational groups, similar to a recent linked data study [20]. Upper non-manual workers (MNG and PRF) had lower SMR of suicide and CCVD than other groups. However, as a result of separately analyzing MNG and PRF, the SMR of MNG was the second highest in suicide and the first in CCVD (Appendix B).

In Europe, the increase in mortality associated with occupations of low socioeconomic status appears to have been directly affected by socioeconomic inequality [17,19]. However, socioeconomic status seems to be insufficient to explain the mortality difference by occupation in Korea after 2010. The economic crisis in Korea may have increased the work-related stress of middle managers, which can influence high suicide mortality [32]. MNG who have died by suicide in Korea experienced various work-related stresses, mainly related to excessive responsibilities compared to other occupations [7,8]. MNG are divided into senior management positions representing institutions or companies and management positions including middle managers within the companies [33]. In Korea, there was a significant decrease in the number of jobs centered on middle management positions between 2008 and 2015, which indicates that restructuring due to the economic crisis may have mainly impacted the middle management positions [34]. A recent study comparing mortality inequality by occupation in Europe, Japan, and Korea speculated that the working conditions and social culture in Korea and Japan that cause overwork of MNG are different from those in Europe [19].

Most European countries have reported that the 2008 economic crisis had a negative impact on health, but the results for gender vary by country [35,36]. While the United Kingdom did not find a direct link between the economic crisis and women’s suicide [35], Greece had a high suicide rate among women in management positions in 2000–2009 [11]. A previous Korean study has reported that women are less affected by the economic downturn than men in Korea, with a decrease in the suicide rate among women and an increase in the suicide rate among men [21]. However, our study results show that SMR of female suicide and CCVD in MNG increased sharply after the 2008 global economic crisis. Suicide was highest in the 1997 economic crisis as well. The high SMR of female MNG may indicate that national economic difficulties and social changes affect female MNG more directly than females in other occupations in the Korean labor market. The promotion of Korean women to managerial positions is rare, despite their having a high level of education similar to that of men [37]. The high mortality rate of female MNG may be because of overwork, increased responsibilities, preferential dismissal, decreased employment rate, and decreased managerial population due to the economic crisis [34,38]. Therefore, for female MNG with the highest mortality risk, a fundamental and long-term policy approach is essential for resolving occupational gender discrimination and preventing overwork. It is a part that requires a lot of research and social attention for preventive intervention. 

Although the SMR of MNG increased rapidly for women after 2008 and for men after 2012, even though SFF withdrew from the top spot, the SMR of SFF is still high in suicide and CCVD [19,20,31]. SFF is also known as an occupation most affected by the economic crisis, with higher mortality [11,19,39]. The causes are presumed to be overwork due to a continuous decline in the labor force, financial difficulties due to changes in the industrial structure, and difficulties in accessing medical services [19,40,41]. Additionally, work environments with physical risk factors are known to be more stressful for female workers than for males, which can lead to mortality risk factors [29].

The SMR of MNG and SFF was higher than that of other occupations and significant, but the PMR was low or insignificant, which was more pronounced in women than in men. This may be an effect of the decreasing population of the relevant occupation. Suicide, CCVD deaths, and total deaths in female MNG surged from 2009 to 2017, while the population of female MNG continued to decline from 2012 to 2017. SMRs of suicide and CCVD for female MNG were noticeably higher than for males. However, the PMR was low or not significant, which may have been overestimated, mainly due to the relatively small number of female MNG in the population [29]. Similarly, male SFF’s suicide and CCVD deaths decreased, and their population is also rapidly decreasing. Although both suicide and CCVD had high SMR, the PMR was low or insignificant for SFF.

This study had several limitations. First, the results are not representative of suicide deaths for the entire working-age population in Korea. To compare the mortality by occupation, we analyzed the data of employed people only. The Armed Forces and other unclassified people among the employed population, unemployed people among the economically active population, and the economically inactive population were excluded from the study. Second, although the linkage table was followed for reclassification from the old version to the 7th version of KSCO by the Korean Statistical Classification of Statistics Korea [33], some reclassification of occupations may not be accurate. Third, changes in the CDS regarding recording the occupation of the deceased may have affected the change in mortality by occupation over a 24-year period. Around the year 2000, the method of recording the occupation was changed from “occupation before death” to “occupation at the time of illness or accident”. However, according to the present results, the pattern of mortality by occupation did not change significantly around 2000. Moreover, after 2018, the CDS used administrative data to determine the deceased’s occupation, as this information was excluded from the death certificate. Although these changes seem to have influenced the mortality trend by occupation after 2018, it is noteworthy that the mortality of female MNG continues to be the highest. Finally, SMR and PMR have a disadvantage in that they do not provide information on the causal relationship between occupation and mortality. Additionally, since SMR was calculated using unlinked data, there may be a numerator–denominator bias in mortality estimates, though the PMR was analyzed to compensate for this bias. In order to understand the causes of mortality by occupation, further research linking CDS with data that have variables that can affect suicide in economically active populations is needed.

## 5. Conclusions

This study investigates the trends and changes in occupational mortality over 24 years in Korea by analyzing the SMR and PMR of suicide and CCVD, which are types of overwork-related deaths. After the global economic crisis, both male and female MNG had the highest mortality ratios for suicide and CCVD, whereas the SMR of PRF was consistently low. As MNG and PRF are known to have similarly high socioeconomic status, previous studies analyzed the mortality by sorting them into the same group. However, we found differences in mortality between MNG and PRF, which suggests that they should be analyzed separately. Female MNG had the highest SMR and low PMR compared to other occupations, which may be due to decreased population, increased responsibilities, and priority layoffs during the economic crisis. Occupational differences between suicide and CCVD mortality highlight the need for management of work-related stress, early treatment, and preventive intervention in the workplace, especially in high-mortality occupations.

## Figures and Tables

**Table 1:** SMR and PMR of male suicide by occupation, every 3 years.

	**1997–1999**			**2000–2002**			**2003–2005**			**2006–2008**		
	**N**	**SMR (CI)**	**PMR (CI)**	**N**	**SMR (CI)**	**PMR (CI)**	**N**	**SMR (CI)**	**PMR (CI)**	**N**	**SMR (CI)**	**PMR (CI)**
Managers (MNG)	75	0.24	0.81	61	0.19	0.71	96	0.20	0.89	188	0.50	1.14
		(0.18–0.29)	(0.63–1.00)		(0.14–0.24)	(0.53–0.88)		(0.16–0.24)	(0.71–1.07)		(0.43–0.57)	(0.97–1.30)
Professionals and related (PRF)	640	0.49	0.82	543	0.48	0.79	910	0.70	0.83	806	0.67	0.85
		(0.45–0.52)	(0.75–0.88)		(0.44–0.52)	(0.73–0.86)		(0.65–0.75)	(0.78–0.88)		(0.63–0.72)	(0.79–0.90)
Clerks (CLK)	769	1.14	0.78	748	1.26	0.80	1199	1.12	0.84	1305	1.42	0.89
		(1.06–1.22)	(0.73–0.84)		(1.17–1.35)	(0.74–0.86)		(1.05–1.18)	(0.80–0.89)		(1.34–1.50)	(0.79–0.90)
Service and sales (SSL)	1442	1.20	1.11	1453	1.37	1.03	2408	1.59	1.14	2087	1.63	1.11
		(1.14–1.26)	(1.05–1.17)		(1.30–1.44)	(0.97–1.08)		(1.52–1.65)	(1.10–1.19)		(1.56–1.70)	(1.06–1.16)
Skilled agricultural, forestry, and fishery (SFF)	1723	2.63	1.00	1744	2.90	1.17	1814	2.67	1.05	1274	2.83	1.08
		(2.50–2.75)	(0.95–1.04)		(2.77–3.04)	(1.11–1.22)		(2.55–2.79)	(1.00–1.10)		(2.67–2.98)	(1.02–1.14)
Craft and related trades (CRT)	1076	0.88	0.97	701	0.65	0.95	884	0.64	0.95	708	0.59	0.93
		(0.83–0.94)	(0.91–1.02)		(0.60–0.70)	(0.88–1.02)		(0.59–0.68)	(0.89–1.02)		(0.55–0.64)	(0.86–0.99)
Equipment, machine operating, and assembling (QMS)	625	0.53	1.25	361	0.34	1.06	484	0.31	1.12	412	0.29	1.13
		(0.49–0.57)	(1.15–1.35)		(0.30–0.37)	(0.96–1.17)		(0.28–0.34)	(1.02–1.22)		(0.27–0.32)	(1.02–1.24)
Elementary (LMT)	871	1.33	1.20	857	1.38	1.10	1096	1.21	1.02	851	1.06	0.99
		(1.24–1.42)	(1.12–1.38)		(1.29–1.47)	(1.03–1.18)		(1.14–1.28)	(0.96–1.08)		(0.98–1.13)	(0.93–1.06)
Total	7221			6468			8891			7631		
	**2009–2011**			**2012–2014**			**2015–2017**			**2018–2020**		
	**N**	**SMR (CI)**	**PMR (CI)**	**N**	**SMR (CI)**	**PMR (CI)**	**N**	**SMR (CI)**	**PMR (CI)**	**N**	**SMR (CI)**	**PMR (CI)**
Managers (MNG)	610	1.34	1.08	968	2.75	1.08	1007	4.01	1.07	283	1.03	0.64
		(1.24–1.45)	(0.99–1.16)		(2.58–2.92)	(1.01–1.15)		(3.76–4.26)	(1.00–1.14)		(0.91–1.16)	(0.57–0.72)
Professionals and related (PRF)	1181	0.63	0.89	1372	0.66	0.96	1434	0.73	0.97	965	0.46	0.98
		(0.60–0.67)	(0.84–0.94)		(0.62–0.69)	(0.91–1.01)		(0.69–0.77)	(0.92–1.02)		(0.43–0.49)	(0.92–1.04)
Clerks (CLK)	2240	1.50	0.96	1675	0.94	0.97	1301	0.75	0.93	2817	1.56	1.35
		(1.44–1.56)	(0.92–1.00)		(0.90–0.99)	(0.93–1.02)		(0.71–0.79)	(0.87–0.98)		(1.50–1.61)	(1.30–1.40)
Service and sales (SSL)	2726	1.65	1.14	2938	1.65	1.18	2623	1.63	1.13	2240	1.33	1.08
		(1.59–1.71)	(1.10–1.19)		(1.59–1.71)	(1.14–1.22)		(1.57–1.70)	(1.09–1.17)		(1.28–1.39)	(1.04–1.13)
Skilled agricultural, forestry, and fishery (SFF)	1190	2.23	0.98	903	1.90	0.86	664	1.78	0.81	445	1.14	0.96
		(2.10–2.35)	(0.92–1.03)		(1.77–2.02)	(0.81–0.92)		(1.64–1.91)	(0.75–0.87)		(1.04–1.25)	(0.87–1.05)
Craft and related trades (CRT)	981	0.62	0.88	1001	0.59	0.87	942	0.59	0.98	349	0.22	0.64
		(0.58–0.65)	(0.83–0.94)		(0.55–0.63)	(0.82–0.92)		(0.55–0.62)	(0.91–1.04)		(0.20–0.24)	(0.57–0.71)
Equipment, machine operating, and assembling (QMS)	556	0.28	0.97	785	0.35	0.97	788	0.37	0.92	915	0.46	0.90
		(0.26–0.30)	(0.89–1.05)		(0.33–0.38)	(0.9–1.04)		(0.35–0.40)	(0.86–0.99)		(0.43–0.49)	(0.85–0.96)
Elementary (LMT)	1190	1.08	1.03	1810	1.72	0.95	1890	1.88	1.01	2880	2.75	0.88
		(1.02–1.15)	(0.97–1.08)		(1.64–1.80)	(0.91–1.00)		(1.79–1.96)	(0.97–1.06)		(2.65–2.85)	(0.85–0.91)
Total	10,674			11,452			10,649			10,894		

N—observed death; SMR—indirect standardized mortality ratio; PMR—standardized proportional mortality ratio; CI—95% confidence intervals.

**Table 2:** SMR and PMR of female suicide by occupation, every 3 years.

	**1997–1999**			**2000–2002**			**2003–2005**			**2006–2008**		
	**N**	**SMR (CI)**	**PMR (CI)**	**N**	**SMR (CI)**	**PMR (CI)**	**N**	**SMR (CI)**	**PMR (CI)**	**N**	**SMR (CI)**	**PMR (CI)**
Managers (MNG)	12	3.89	1.32	5	1.29	0.85	9	1.15	1.14	21	2.15	0.98
		(1.69–6.10)	(0.57–2.06)		(0.16–2.41)	(0.11–1.60)		(0.40–1.91)	(0.39–1.88)		(1.23–3.07)	(0.56–1.41)
Professionals and related (PRF)	127	0.87	0.86	81	0.65	0.77	163	0.68	0.82	269	0.67	0.88
		(0.72–1.02)	(0.71–1.00)		(0.51–0.79)	(0.60–0.94)		(0.58–0.79)	(0.70–0.95)		(0.59–0.75)	(0.78–0.99)
Clerks (CLK)	106	0.84	0.78	113	1.00	0.72	281	1.23	0.96	443	1.20	0.92
		(0.68–1.01)	(0.63–0.93)		(0.81–1.18)	(0.59–0.85)		(1.08–1.37)	(0.85–1.08)		(1.09–1.31)	(0.83–1.01)
Service and sales (SSL)	331	0.81	1.11	325	0.80	1.03	553	0.96	1.04	696	1.16	1.09
		(0.72–0.89)	(0.99–1.23)		(0.71–0.89)	(0.91–1.14)		(0.88–1.05)	(0.95–1.12)		(1.07–1.25)	(1.01–1.17)
Skilled agricultural, forestry, and fishery (SFF)	345	3.30	1.06	358	3.35	1.27	374	3.45	1.15	259	3.18	1.09
		(2.96–3.65)	(0.95–1.17)		(3.00–3.69)	(1.14–1.40)		(3.10–3.80)	(1.03–1.26)		(2.79–3.57)	(0.96–1.23)
Craft and related trades (CRT)	39	0.48	0.85	30	0.41	0.74	34	0.46	0.75	48	0.75	0.98
		(0.33–0.63)	(0.58–1.11)		(0.26–0.55)	(0.47–1.00)		(0.31–0.61)	(0.46–1.00)		(0.54–0.96)	(0.7–1.26)
Equipment, machine operating, and assembling (QMS)	1	0.03	0.27	13	0.38	1.72	9	0.16	0.78	11	0.17	0.91
		(0.00–0.08)	(0.00–0.80)		(0.17–0.58)	(0.78–2.65)		(0.05–0.26)	(0.27–1.30)		(0.07–0.27)	(0.37–1.45)
Elementary (LMT)	69	0.56	1.07	66	0.52	0.86	98	0.42	0.93	99	0.39	0.99
		(0.43–0.70)	(0.82–1.33)		(0.40–0.65)	(0.65–1.07)		(0.34–0.51)	(0.74–1.11)		(0.1–0.46)	(0.79–1.18)
Total	1030			991			1521			1846		
	**2009–2011**			**2012–2014**			**2015–2017**			**2018–2020**		
	**N**	**SMR (CI)**	**PMR (CI)**	**N**	**SMR (CI)**	**PMR (CI)**	**N**	**SMR (CI)**	**PMR (CI)**	**N**	**SMR (CI)**	**PMR (CI)**
Managers (MNG)	102	7.64	1.06	100	8.65	0.94	97	13.17	0.99	39	3.14	0.60
		(6.16–9.13)	(0.86–1.27)		(6.95–10.34)	(0.76–1.13)		(10.55–15.79)	(0.79–1.19)		(2.15–4.12)	(0.41–0.79)
Professionals and related (PRF)	463	0.71	0.84	447	0.70	0.87	411	0.67	0.85	591	0.87	1.18
		(0.64–0.77)	(0.77–0.92)		(0.64–0.77)	(0.79–0.95)		(0.60–0.73)	(0.77–0.93)		(0.80–0.94)	(1.09–1.28)
Clerks (CLK)	640	1.08	0.90	503	0.88	0.89	413	0.78	0.82	628	1.04	1.12
		(1.00–1.17)	(0.83–0.97)		(0.80–0.96)	(0.81–0.96)		(0.71–0.86)	(0.74–0.90)		(0.96–1.12)	(1.03–1.21)
Service and sales (SSL)	1025	1.33	1.19	1011	1.39	1.18	1036	1.53	1.24	840	1.24	1.06
		(1.25–1.41)	(1.12–1.27)		(1.31–1.48)	(1.11–1.25)		(1.44–1.63)	(1.16–1.31)		(1.16–1.33)	(0.99–1.13)
Skilled agricultural, forestry, and fishery (SFF)	215	2.72	1.10	134	2.08	0.97	79	1.78	0.76	62	1.28	0.74
		(2.35–3.08)	(0.95–1.25)		(1.73–2.43)	(0.81–1.13)		(1.38–2.17)	(0.60–0.93)		(0.96–1.60)	(0.55–0.92)
Craft and related trades (CRT)	51	0.65	0.89	67	0.94	1.00	60	0.93	1.05	15	0.25	0.55
		(0.47–0.82)	(0.64–1.13)		(0.72–1.17)	(0.76–1.24)		(0.69–1.16)	(0.78–1.31)		(0.12–0.38)	(0.27–0.82)
Equipment, machine operating, and assembling (QMS)	6	0.07	0.35	21	0.21	0.75	23	0.26	0.86	35	0.43	0.48
		(0.01–0.13)	(0.07–0.63)		(0.12–0.30)	(0.43–1.07)		(0.16–0.37)	(0.51–1.21)		(0.29–0.57)	(0.32–0.64)
Elementary (LMT)	110	0.32	0.87	212	0.67	0.99	190	0.67	0.95	222	0.83	0.67
		(0.26–0.38)	(0.71–1.03)		(0.58–0.76)	(0.85–1.12)		(0.57–0.76)	(0.81–1.08)		(0.72–0.94)	(0.58–0.76)
Total	2612			2495			2309			2432		

N—observed death; SMR—indirect standardized mortality ratio; PMR—standardized proportional mortality ratio; CI—95% confidence intervals.

**Table 3:** SMR and PMR of male CCVD death by occupation, every 3 years.

	**1997–1999**			**2000–2002**			**2003–2005**			**2006–2008**		
	**N**	**SMR (CI)**	**PMR (CI)**	**N**	**SMR (CI)**	**PMR (CI)**	**N**	**SMR (CI)**	**PMR (CI)**	**N**	**SMR (CI)**	**PMR (CI)**
Managers (MNG)	237	0.27	1.08	264	0.30	1.20	208	0.28	1.21	243	0.45	1.05
		(0.24–0.31)	(0.94–1.21)		(0.26–0.33)	(1.05–1.34)		(0.24–0.32)	(1.05–1.38)		(0.39–0.50)	(0.92–1.19)
Professionals and related (PRF)	1614	0.80	1.13	1388	0.74	1.07	1480	1.05	1.08	1180	0.97	1.06
		(0.76–0.84)	(1.08–1.19)		(0.70–0.78)	(1.01–1.12)		(0.99–1.10)	(1.02–1.13)		(0.91–1.02)	(1.00–1.12)
Clerks (CLK)	1799	2.19	1.21	1759	2.10	1.17	1765	1.61	1.14	1622	1.87	1.14
		(2.09–2.29)	(1.16–1.27)		(2.00–2.19)	(1.11–1.22)		(1.54–1.69)	(1.09–1.20)		(1.78–1.96)	(1.08–1.19)
Service and sales (SSL)	2923	1.26	1.19	3318	1.53	1.16	3233	1.60	1.15	2587	1.65	1.10
		(1.21–1.30)	(1.15–1.24)		(1.48–1.58)	(1.12–1.20)		(1.54–1.65)	(1.11–1.19)		(1.59–1.72)	(1.06–1.14)
Skilled agricultural, forestry, and fishery (SFF)	4459	1.63	0.81	3644	1.60	0.80	2372	1.60	0.73	1576	1.83	0.77
		(1.58–1.68)	(0.79–0.83)		(1.55–1.65)	(0.77–0.82)		(1.53–1.66)	(0.70–0.76)		(1.74–1.92)	(0.73–0.81)
Craft and related trades (CRT)	1681	0.84	0.97	1344	0.68	1.01	1161	0.65	1.02	841	0.59	0.95
		(0.80–0.88)	(0.93–1.02)		(0.64–0.71)	(0.96–1.06)		(0.61–0.68)	(0.96–1.08)		(0.55–0.63)	(0.89–1.02)
Equipment, machine operating and assembling (QMS)	847	0.40	1.18	636	0.30	1.11	564	0.27	1.10	456	0.26	1.11
		(0.37–0.42)	(1.10–1.26)		(0.28–0.33)	(1.02–1.20)		(0.25–0.29)	(1.01–1.19)		(0.23–0.28)	(1.01–1.21)
Elementary (LMT)	1527	0.71	0.98	1701	0.88	1.01	1547	0.92	1.02	1067	0.82	0.96
		(0.67–0.75)	(0.93–1.03)		(0.84–0.92)	(0.96–1.06)		(0.87–0.97)	(0.97–1.07)		(0.77–0.87)	(0.90–1.01)
Total	15,087			14,054			12,330			9572		
	**2009–2011**			**2012–2014**			**2015–2017**			**2018–2020**		
	**N**	**SMR (CI)**	**PMR (CI)**	**N**	**SMR (CI)**	**PMR (CI)**	**N**	**SMR (CI)**	**PMR (CI)**	**N**	**SMR (CI)**	**PMR (CI)**
Managers (MNG)	581	1.32	1.16	809	2.39	1.09	850	3.16	1.00	414	1.52	1.17
		(1.21–1.42)	(1.07–1.26)		(2.23–2.56)	(1.01–1.16)		(2.95–3.37)	(0.93–1.07)		(1.37–1.66)	(1.06–1.29)
Professionals and related (PRF)	1062	0.90	1.05	1059	0.83	1.02	1177	0.87	0.99	687	0.54	0.99
		(0.85–0.96)	(0.99–1.11)		(0.78–0.88)	(0.96–1.09)		(0.82–0.92)	(0.94–1.05)		(0.50–0.58)	(0.92–1.07)
Clerks (CLK)	1504	1.68	1.06	1099	1.05	1.06	1006	0.84	1.08	1226	1.07	1.06
		(1.59–1.76)	(1.01–1.12)		(0.98–1.11)	(1.00–1.12)		(0.79–0.89)	(1.01–1.15)		(1.01–1.13)	(1.00–1.12)
Service and sales (SSL)	2076	1.62	1.07	2010	1.51	1.06	2143	1.66	1.13	1513	1.28	1.06
		(1.55–1.69)	(1.02–1.11)		(1.45–1.58)	(1.01–1.11)		(1.59–1.73)	(1.08–1.18)		(1.21–1.34)	(1.01–1.12)
Skilled agricultural, forestry, and fishery (SFF)	1090	1.63	0.78	924	1.55	0.77	777	1.55	0.76	378	0.80	0.73
		(1.53–1.73)	(0.73–0.82)		(1.45–1.65)	(0.72–0.82)		(1.44–1.66)	(0.70–0.81)		(0.72–0.88)	(0.66–0.81)
Craft and related trades (CRT)	851	0.66	0.99	857	0.62	1.01	736	0.50	0.91	407	0.31	0.94
		(0.62–0.70)	(0.92–1.06)		(0.58–0.66)	(0.94–1.07)		(0.46–0.53)	(0.85–0.98)		(0.28–0.34)	(0.85–1.03)
Equipment, machine operating, and assembling (QMS)	483	0.28	1.09	565	0.29	0.99	694	0.34	1.01	957	0.56	1.07
		(0.25–0.30)	(0.99–1.18)		(0.27–0.31)	(0.91–1.08)		(0.32–0.37)	(0.94–1.09)		(0.53–0.60)	(1.00–1.14)
Elementary (LMT)	961	0.85	0.93	1676	1.57	1.01	1838	1.69	1.00	2753	2.83	0.96
		(0.80–0.91)	(0.88–0.99)		(1.50–1.65)	(0.96–1.05)		(1.61–1.77)	(0.95–1.04)		(2.73–2.94)	(0.92–1.00)
Total	8608			8999			9221			8335		

CCVD—cerebro-cardiovascular diseases; N—observed death; SMR—indirect standardized mortality ratio; PMR—standardized proportional mortality ratio; CI—95% confidence intervals.

**Table 4:** SMR and PMR of female CCVD death by occupation, every 3 years.

	**1997–1999**			**2000–2002**			**2003–2005**			**2006–2008**		
	**N**	**SMR (CI)**	**PMR (CI)**	**N**	**SMR (CI)**	**PMR (CI)**	**N**	**SMR (CI)**	**PMR (CI)**	**N**	**SMR (CI)**	**PMR (CI)**
Managers (MNG)	15	1.05	0.91	7	0.45	0.74	6	0.64	0.66	12	1.40	0.72
		(0.52–1.58)	(0.45–1.36)		(0.12–0.79)	(0.19–1.29)		(0.13–1.16)	(0.13–1.19)		(0.61–2.20)	(0.31–1.12)
Professionals and related (PRF)	143	1.26	0.81	103	0.94	0.83	106	0.92	0.82	104	0.89	0.75
		(1.05–1.46)	(0.68–0.94)		(0.76–1.12)	(0.67–0.99)		(0.74–1.09)	(0.66–0.97)		(0.72–1.07)	(0.60–0.89)
Clerks (CLK)	129	1.46	1.10	152	1.66	0.93	135	1.41	0.87	149	1.61	0.91
		(1.21–1.71)	(0.91–1.29)		(1.39–1.92)	(0.78–1.08)		(1.17–1.65)	(0.73–1.02)		(1.36–1.87)	(0.76–1.05)
Service and sales (SSL)	640	0.57	1.11	679	0.69	1.14	641	0.87	1.16	529	0.96	1.12
		(0.53–0.62)	(1.03–1.20)		(0.64–0.74)	(1.05–1.23)		(0.80–0.94)	(1.07–1.25)		(0.88–1.04)	(1.02–1.21)
Skilled agricultural, forestry, and fishery (SFF)	1730	2.12	0.96	1186	1.95	0.91	727	2.24	0.88	420	2.58	0.93
		(2.02–2.22)	(0.91–1.00)		(1.84–2.06)	(0.86–0.96)		(2.07–2.40)	(0.82–0.95)		(2.33–2.82)	(0.84–1.02)
Craft and related trades (CRT)	86	0.39	1.05	107	0.59	1.08	65	0.66	1.27	47	0.67	1.30
		(0.31–0.48)	(0.83–1.28)		(0.48–0.70)	(0.88–1.28)		(0.50–0.82)	(0.96–1.58)		(0.48–0.86)	(0.93–1.68)
Equipment, machine operating, and assembling (QMS)	7	0.10	0.92	18	0.28	1.14	18	0.30	1.31	9	0.19	1.00
		(0.03–0.17)	(0.24–1.61)		(0.15–0.41)	(0.61–1.67)		(0.16–0.44)	(0.71–1.92)		(0.07–0.31)	(0.35–1.65)
Elementary (LMT)	229	0.42	1.18	255	0.56	1.31	194	0.43	1.21	133	0.38	1.18
		(0.37–0.48)	(1.03–1.33)		(0.49–0.63)	(1.15–1.47)		(0.37–0.49)	(1.04–1.38)		(0.31–0.44)	(0.98–1.39)
Total	2979			2507			1892			1403		
	**2009–2011**			**2012–2014**			**2015–2017**			**2018–2020**		
	**N**	**SMR (CI)**	**PMR (CI)**	**N**	**SMR (CI)**	**PMR (CI)**	**N**	**SMR (CI)**	**PMR (CI)**	**N**	**SMR (CI)**	**PMR (CI)**
Managers (MNG)	59	6.14	1.00	62	7.14	0.92	58	7.93	0.83	48	3.57	1.04
		(4.57–7.71)	(0.74–1.26)		(5.36–8.91)	(0.69–1.15)		(5.89–9.97)	(0.61–1.04)		(2.56–4.58)	(0.74–1.33)
Professionals and related (PRF)	121	0.84	0.65	161	0.86	0.76	185	0.77	0.76	247	0.83	0.88
		(0.69–0.99)	(0.54–0.77)		(0.73–0.99)	(0.65–0.88)		(0.66–0.89)	(0.65–0.86)		(0.72–0.93)	(0.77–0.99)
Clerks (CLK)	176	1.52	0.90	157	1.00	0.88	167	0.85	0.86	237	0.91	0.92
		(1.29–1.74)	(0.77–1.03)		(0.84–1.15)	(0.74–1.02)		(0.72–0.98)	(0.73–0.99)		(0.79–1.02)	(0.80–1.04)
Service and sales (SSL)	506	1.01	1.10	558	1.06	1.08	665	1.18	1.12	690	1.02	1.03
		(0.92–1.10)	(1.00–1.20)		(0.98–1.15)	(0.99–1.17)		(1.09–1.27)	(1.03–1.20)		(0.94–1.09)	(0.95–1.11)
Skilled agricultural, forestry, and fishery (SFF)	250	2.31	0.93	183	2.03	0.93	135	1.95	0.84	147	1.61	0.97
		(2.03–2.60)	(0.81–1.04)		(1.74–2.32)	(0.80–1.07)		(1.62–2.28)	(0.70–0.98)		(1.35–1.87)	(0.81–1.13)
Craft and related trades (CRT)	36	0.57	1.11	36	0.59	0.88	29	0.45	0.68	23	0.32	0.81
		(0.38–0.76)	(0.74–1.47)		(0.40–0.79)	(0.60–1.17)		(0.29–0.61)	(0.44–0.93)		(0.19–0.46)	(0.48–1.13)
Equipment, machine operating, and assembling (QMS)	22	0.46	2.04	20	0.34	1.32	29	0.47	1.59	61	0.88	0.93
		(0.27–0.65)	(1.19–2.89)		(0.19–0.49)	(0.74–1.90)		(0.30–0.64)	(1.01–2.16)		(0.66–1.10)	(0.70–1.17)
Elementary (LMT)	159	0.47	1.38	239	0.73	1.26	261	0.79	1.28	388	1.08	1.14
		(0.39–0.54)	(1.17–1.60)		(0.64–0.82)	(1.10–1.42)		(0.70–0.89)	(1.13–1.44)		(0.97–1.19)	(1.03–1.26)
Total	1329			1416			1529			1841		

CCVD—cerebro-cardiovascular diseases; N—observed death; SMR—indirect standardized mortality ratio; PMR—standardized proportional mortality ratio; CI—95% confidence intervals.

## Data Availability

The Causes of Death Statistics and Economically Active Population Survey used in this analysis can be freely accessed from MDIS (https://mdis.kostat.go.kr) (accessed on 21 December 2021) of Statistics Korea.

## Appendix A

**Table A1:** SMR and PMR of suicide and CCVD by occupation for 24 years (1997–2020).

	Men						Women					
	Suicide			CCVD			Suicide			CCVD		
	N	SMR (CI)	PMR (CI)	N	SMR (CI)	PMR (CI)	N	SMR (CI)	PMR (CI)	N	SMR (CI)	PMR (CI)
Managers (MNG)	3288	1.15	1.26	3606	0.91	1.05	385	5.59	1.13	267	3.01	0.79
		(1.11–1.19)	(1.21–1.30)		(0.88–0.94)	(1.02–1.09)		(5.04–6.15)	(1.02–1.24)		(2.65–3.37)	(0.70–0.89)
Professionals and related (PRF)	7851	0.61	0.91	9647	0.81	1.05	2552	0.78	0.97	1170	0.78	0.72
		(0.59–0.62)	(0.89–0.93)		(0.79–0.82)	(1.03–1.08)		(0.75–0.81)	(0.93–1.01)		(0.73–0.82)	(0.68–0.76)
clerks (CLK)	12,054	1.22	1.02	11,780	1.32	1.12	3127	1.08	0.98	1302	1.04	0.86
		(1.19–1.24)	(1.01–1.04)		(1.30–1.35)	(1.10–1.14)		(1.04–1.11)	(0.94–1.01)		(0.98–1.09)	(0.81–0.91)
Service and sales (SSL)	17,917	1.52	1.14	19,803	1.52	1.12	5817	1.14	1.15	4908	0.86	1.06
		(1.50–1.54)	(1.12–1.15)		(1.50–1.54)	(1.11–1.14)		(1.11–1.17)	(1.12–1.18)		(0.84–0.89)	(1.03–1.09)
Skilled agricultural, forestry, and fishery (SFF)	9757	2.33	0.82	15,220	1.96	0.81	1826	2.63	0.84	4778	2.85	1.05
		(2.28–2.37)	(0.80–0.84)		(1.93–1.99)	(0.80–0.82)		(2.51–2.75)	(0.80–0.87)		(2.77–2.93)	(1.02–1.08)
Craft and related trades (CRT)	6642	0.58	0.83	7878	0.60	0.98	344	0.52	0.79	429	0.56	1.08
		(0.56–0.59)	(0.81–0.85)		(0.59–0.62)	(0.96–1.00)		(0.47–0.58)	(0.71–0.88)		(0.51–0.61)	(0.98–1.18)
Equipment, machine operating, and assembling (QMS)	4926	0.36	1.05	5202	0.31	1.05	119	0.21	0.70	184	0.36	1.00
		(0.35–0.37)	(1.02–1.08)		(0.31–0.32)	(1.02–1.08)		(0.18–0.25)	(0.58–0.83)		(0.31–0.41)	(0.86–1.14)
Elementary (LMT)	11,445	1.60	1.08	13,070	1.19	0.95	1066	0.54	0.88	1858	0.55	1.13
		(1.57–1.63)	(1.06–1.10)		(1.17–1.21)	(0.94–097)		(0.50–0.57)	(0.83–0.94)		(0.52–0.57)	(1.08–1.18)
Total	73,880			86,206			15,236			14,896		

CCVD—cerebro-cardiovascular diseases; N—observed death; SMR—indirect standardized mortality ratio; PMR—standardized proportional mortality ratio; CI—95% confidence intervals.

## Appendix B

**Table A2:** Comparison of SMR by occupational groups in men, 2007–2018.

		Suicide				CCVD			
4 Groups	8 Groups	4 Groups		8 Groups		4 Groups		8 Groups	
		N	SMR (CI)	N	SMR (CI)	N	SMR (CI)	N	SMR (CI)
Upper non–manual	Managers (MNG)	6678	0.93	2602	1.90	6616	1.06	2506	1.76
			(0.91–0.95)		(1.83–1.97)		(1.04–1.09)		(1.69–1.82)
	Professionals and related (PRF)			4076	0.70			4110	0.86
					(0.68–0.72)				(0.83–0.88)
Lower non–manual	Clerks (CLK)	13,933	1.37	5242	1.07	12,701	1.41	4783	1.22
			(1.34–1.39)		(1.04–1.10)		(1.39–1.44)		(1.18–1.25)
	Service and sales (SSL)			8691	1.64			7918	1.56
					(1.60–1.67)				(1.53–1.60)
Skilled agricultural, forestry, and fishery	Skilled agricultural, forestry, and fishery (SFF)	3600	2.08	3600	2.08	3858	1.63	3858	1.63
			(2.01–2.15)		(2.01–2.15)		(1.58–1.68)		(1.58–1.68)
Manual	Craft and related trades (CRT)	11,173	0.69	2989	0.54	11,380	0.67	3047	0.57
			(0.67–0.70)		(0.52–0.56)		(0.66–0.68)		(0.55–0.59)
	Equipment, machine operating, and assembling (QMS)			2394	0.34			2349	0.32
					(0.32–0.35)				(0.31–0.33)
	Elementary (LMT)			5790	1.60			5984	1.39
					(1.56–1.64)				(1.35–1.42)
Total		35,384		35,384		34,555		34,555	

CCVD—cerebro-cardiovascular diseases; N—observed death; SMR—indirect standardized mortality ratio; PMR—standardized proportional mortality ratio; CI—95% confidence intervals.
